# A “Time-Kill” Study to Assess the Effect of Colistin on Gram-Negative Strains: An Experimental Study for Developing an Indigenous Microfluidics-Based Antimicrobial Susceptibility Testing Device

**DOI:** 10.7759/cureus.85321

**Published:** 2025-06-04

**Authors:** Ehsaas Bajaj, Ayush Gupta, Shashank Purwar, Shrutika Pundir, Sukhmanjit S Brar

**Affiliations:** 1 Microbiology, All India Institute of Medical Sciences, Bhopal, Bhopal, IND

**Keywords:** antibiotic resistance, cell-viability assay, colistin, microbial sensitivity test, time-kill assay

## Abstract

The emergence of infections caused by multidrug-resistant gram-negative strains has necessitated the reuse of polymyxins, which are repurposed antimicrobials due to their initial toxicity profile. However, the present antimicrobial susceptibility testing (AST) methods against them are challenging to perform or need validation. Through this study, we sought to determine how early we can differentiate between the susceptible and resistant gram-negative strains when exposed to a single breakpoint concentration of antibiotic, as expected from a microfluidics-based AST. We used time-kill assay and cell-viability assay to image the organisms and draw a parallel to the principle of microfluidics-based assay.

Reference and clinical strains of Enterobacterales and *Pseudomonas aeruginosa* of a particular minimum inhibitory concentration (MIC) value were selected for the study after initially screening by the colistin broth disk elution method and confirmed by colistin broth microdilution (BMD). For *Acinetobacter baumannii* complex, only a BMD test was used to determine the MIC. Ten strains of varying MICs, ranging from ≤0.5 to ≥16 μg/mL, were selected for time-kill assay against colistin using acridine orange dye to obtain each strain's total cell count of antibiotic-exposed and non-exposed populations at different time points. These strains were then subjected to the spread-plate method for viability counts at various time points.

We obtained 192 unique measurements from 48 colistin-sensitive replicates belonging to 8 strains with MICs ranging from ≤0.5 to 2 μg/mL at different time points. We found that the log count values were statistically lower in the colistin-exposed group at 2, 4, and 8 hours by both time-kill and spread-plate assays. We concluded that it is possible to get AST results against colistin as early as 2 hours if imaging techniques are used for visualizing the bacteria in a microfluidics-based AST device.

## Introduction

In the current “bad bugs, no drugs” era, only a few antibiotics are approved for treating infections caused by multidrug-resistant organisms. Consequently, previously overlooked classes of drugs such as polymyxins are utilized as one of the last-resort options [1]. They are employed in the treatment of serious infections caused by carbapenem-resistant Enterobacteriaceae, carbapenem-resistant *Pseudomonas aeruginosa*, and carbapenem-resistant *Acinetobacter baumannii* complex [2,3].

According to the Antimicrobial Resistance and Surveillance Network Report 2023, the susceptibility rate of *Klebsiella pneumoniae* to carbapenems among isolates from all specimens except urine and feces stands at 34.2-37.7% [4]. Similarly, *A. baumannii* exhibits a susceptibility rate of 10.3-12.1%, *P. aeruginosa* exhibits a susceptibility rate of 61.5-65.5%, and *Escherichia coli *shows a susceptibility rate of 59.8-66% [4]. These data suggest a rising trend in resistance rates against a commonly used antibiotic class for managing life-threatening infections. The same surveillance report revealed colistin susceptibility rates, using the broth microdilution (BMD) method, to be 96.2% for *K. pneumoniae*, 96.3% for *P. aeruginosa*, 98.9% for *A. baumannii*, and 99.3% for *E. coli* [4].

The Kirby-Bauer disk diffusion method and automated systems such as Vitek® 2 (bioMerieux, Marcy-l’Etoile, France) are the standard methods for performing antimicrobial susceptibility testing (AST) in routine diagnostic laboratories [5]. However, BMD is the gold standard for testing colistin sensitivity. In India, only selected reference laboratories perform BMD due to its labor-intensive nature, taking 16-24 hours to yield susceptibility results [6]. The Clinical & Laboratory Standards Institute (CLSI) recommends colistin broth disk elution (CBDE) and colistin agar dilution (CAD) for colistin susceptibility testing [7]. However, there is practically no reduction in testing time as they require overnight incubation. Consequently, physicians often administer polymyxin drugs empirically while awaiting susceptibility results, leading to their misuse and outbreaks of colistin-resistant infections [2,8-10]. These methods are also not approved for *A. baumannii* complex infections, which are frequently multidrug-resistant [7,11]. Therefore, there is an urgent need to develop accelerated assays to determine antimicrobial susceptibility, especially for last-resort antibiotics such as the polymyxin class of drugs.

Microfluidics-based AST is a miniaturized, reliable, and rapid method of testing for the susceptibility of various organisms subjected to different antibiotics by single-cell imaging in microfluidic channels [12]. We aimed to provide the basis for developing in-house microfluidics-based devices for colistin AST assay. We hypothesized that colistin-sensitive organisms would exhibit differing growth rates during their early interaction with colistin in colistin-exposed and non-exposed conditions. We used fluorescence microscopy in time-kill and cell-viability assays to simulate and draw parallels with time-dependent bacterial killing observed in microfluidics-based systems. This is a preliminary in vitro study with a broader goal of developing an imaging algorithm for creating an indigenous microfluidics-based device capable of predicting sensitivity for colistin within 2 to 8 hours.

## Materials and methods

Study design and setting

This laboratory-based experimental study was conducted from August 2022 to October 2022 in the bacteriology laboratory of the Department of Microbiology, All India Institute of Medical Sciences (AIIMS) Bhopal, Bhopal, Madhya Pradesh, India. We used clinical and standard strains of Enterobacterales, *P. aeruginosa*, and *A. baumannii* with varying minimum inhibitory concentrations (MICs).

Screening and selection of strains

**Figure 1:**
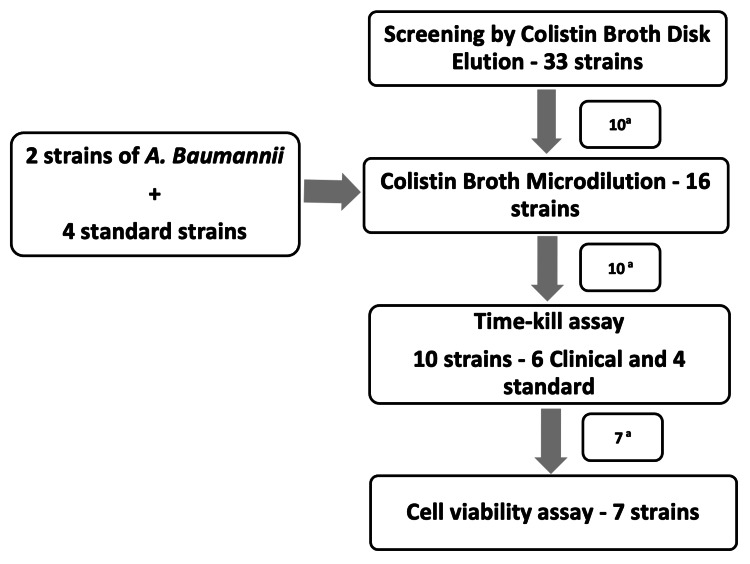
Flow of work ^a^ Strains were selected based on the consistency of values

As described in Figure 1, 33 clinical strains of *E. coli*, *K. pneumoniae*, and* P. aeruginosa* were initially screened using the CBDE method to determine their MIC against colistin. Of these, 10 clinical strains were selected for undertaking the colistin BMD to determine the final MIC. For the four standard ATCC® strains, namely, *E. coli *25922, *E. coli* BAA 3170, *P. aeruginosa* 27853, and *Proteus mirabilis* 12453, and two clinical strains of *A. baumannii*, the MIC was determined directly using the BMD method, as the CBDE method is not validated for *A. baumannii*. Of these 16 strains, which were subjected to BMD, we selected 10 strains for the time-kill assay so that a minimum of two strains were present in each MIC category, which were <0.5 μg/mL, 1 to ≤2 μg/mL, and >2 μg/mL. Those clinical strains that gave consistent MIC values in repeat testing were preferentially selected. This was followed by a time-kill and cell-viability assay to determine the growth curve against a single colistin breakpoint concentration value of 4 µg/mL.

Colistin broth disk elution

We performed CBDE according to CLSI M100 guidelines [7]. Briefly, for the test procedure, we prepared Cation-adjusted Mueller Hinton Broth (CAMHB) (HiMedia®, Mumbai, India) tubes containing 10 mL of media. For testing each isolate, we took four CAMHB tubes and added 1, 2, and 4 colistin sulphate disks (10 µg) (HiMedia® Mumbai, India) to the three tubes. They were then vortexed, and the disks were allowed to elute for 30-60 minutes, giving a concentration of 1, 2, and 4 µg/mL of colistin, respectively. In the fourth tube, no colistin disk was added to serve as “growth control” for the tested isolate. Bacterial inoculum was prepared by transferring three to five colonies from the plated media after overnight growth to sterile saline, and turbidity was adjusted to 0.5 McFarland using Vitek Densichek® (bioMerieux, Marcy-l'Étoile, France). To each tube, 50 μL of the inoculum was added within 15 minutes of preparation, and the tubes were subsequently incubated overnight at 35±2°C under aerobic incubation. Purity checks were performed from the growth control tube by subculturing on a nutrient agar plate.

Broth microdilution method

We performed the colistin BMD method as per the NCDC protocol [13] for 16 strains including 10 clinical strains, 2 strains of *A. baumannii*, and 4 ATCC® strains, as shown in Figure 1. The BMD testing for each included isolate was conducted in duplicates on two separate occasions, and only readings displaying a difference of 1-log dilution were considered valid. In discrepancies, the final MIC was determined based on the value occurring at least three times.

Briefly, a primary stock solution of colistin having a concentration of 1,000 µg/mL was prepared based on the potency of colistin sulphate powder (HiMedia®, Mumbai, India) and stored at -20°C in cryovials. From this, a working solution of 64 µg/mL (four times the final drug concentration) was made whenever the test was performed by mixing 936-µL sterile Mueller-Hinton broth (MHB) and 64 µL of stock colistin solution. Appropriate dilutions were made to achieve concentrations of 32, 16, 8, 4, 2, 1, 0.5, 0.25, and 0.125 µg/mL. In each column of the 96-well microtiter plate labeled 1-10, we added 25 µL of these dilutions, ranging from 64 to 0.125 µg/mL. In each well, 50 µL of MHB was added except in columns 11 and 12, wherein 75 µL and 100 µL of MHB were added to serve as growth and media controls, respectively.

Subsequently, bacterial inoculum of each isolate, including *E. coli* ATCC® 25922 and *P. aeruginosa* ATCC® 27853, was prepared by transferring three to five colonies from the plated media, after overnight incubation, to sterile saline, and turbidity was adjusted to 0.5 McFarland using Vitek Densichek®. This suspension was diluted 1:75 times by adding 10 µL to 740 µL of MHB. Then, 25 µL of this diluted bacterial inoculum containing 5 × 10^4^ CFU/mL was added, in duplicate, to respective rows. After adding the bacterial inoculum, the antibiotic concentration in each well decreased by four times from 16 to 0.03 µg/mL in columns 1-10, respectively. Plates were subsequently incubated at 35±2°C under aerobic incubation, and readings were taken at 16-20 hours for Enterobacterales and *P. aeruginosa* and between 20 and 24 hours for *A. baumannii* complex. The lowest colistin concentration inhibiting bacterial growth was taken as the MIC value.

The appropriateness of the inoculum suspension was checked as follows: immediately after adding the bacterial inoculum in the growth control well, 10 µL of suspension was taken and diluted in 10 mL of normal saline. After mixing, 100 µL of suspension was spread over a nutrient agar plate and incubated overnight at 35±2°C. The presence of approximately 50 colonies indicated appropriate inoculum density.

Time-kill assay

The strains selected for the time-kill assay are detailed in Table 1. These strains were subjected to the time-kill to determine the growth curve with a single breakpoint concentration of colistin, i.e., 4 µg/mL. We made a standard inoculum of bacteria with CAMHB for 10 strains. Colistin was added to the test group, and controls were kept antibiotic-free. Appropriate dilutions were made for feasible counting in a Neubauer’s chamber (Marienfeld, Germany). Counting began from a 3-log dilution of a mixture of 0.5 McFarland solution with colistin (concentration: 4 µg/mL), which had to be serially diluted in subsequent time stamps to up to a 9-log dilution. The total bacterial count was done in the Neubauer chamber by counting the number of cells seen with acridine orange dye under a trinocular fluorescent microscope (Labomed LX-400®, Mumbai, India). Graphs were plotted at various incubation points, such as 0, 1, 2, 4, and 8 hours. Each colistin-sensitive strain (8 out of 10 selected) was tested in triplicate for both test and control at each time point.

**Table 1:** MICs obtained by colistin BMD method of selected samples BMD, broth microdilution; MIC, minimum inhibitory concentration

Organism	Source	MIC (µg/mL) (from BMD)
Escherichia coli	ATCC® 25922	Obtained value: 0.5; expected range: 0.25-2
Pseudomonas aeruginosa	Urine	≤0.5
Pseudomonas aeruginosa	ATCC® 27853	Obtained value: 1; expected range: 0.5-4
Klebsiella pneumoniae	Urine	1
*Acinetobacter baumannii* complex	Endotracheal tube	1
*Escherichia coli *mcr-1 strain	ATCC® BAA 3170	Obtained value: 2; expected value: ≤1 to 4
Escherichia coli	Pus	2
Pseudomonas aeruginosa	Pus	2
Klebsiella pneumoniae	Endotracheal tube	≥16
Proteus mirabilis	ATCC® 12453	Obtained value: ≥16; expected value: inherently resistant

Cell-viability assay

The spread-plate method was used for cell-viability testing at the same time intervals of exposure to colistin as used in the time-kill assay, i.e., at 0, 1, 2, 4, and 8 hours simultaneously with the control (non-exposed) group. Bacterial inocula were made from a range of 3-log dilution (~10^6^) to a 9-log dilution (~10^1^) from a 0.5 McFarland solution of bacterial inoculum. All six dilutions were then plated on a single 100-mm Mueller-Hinton agar plate (HiMedia®, Mumbai, India), which was divided into six parts. The amount of inoculum taken was uniform, i.e., 25 µL, which was spread on 1/6th of the agar plate, and six dilutions of a strain were plated on a single plate. This was repeated at 0, 1, 2, 4, and 8 hours for both the test and the control. Colonies were counted after 18-24 hours of incubation. The dilution at which countable colonies (between 30-300) were observed was selected, and an appropriate multiplication factor was used to calculate the viable colony count in terms of CFU/mL. Subsequently, the graph for growth curves was plotted using these values till 8-hour post-exposure, as the microfluidic assay aims to give AST results within this time frame.

Statistical analysis

Data were downloaded in Excel, cleaned, and coded. Data analysis was done using R software version 4.1.2 (R Foundation for Statistical Computing, Vienna, Austria). The continuous variables were summarized as mean and standard deviation when customarily distributed and as median and interquartile range depending on the data distribution. Depending on the distribution, the association between bacterial counts and the presence or absence of antibiotics at different time points was tested using the unpaired t-test/Mann-Whitney U-test. A mixed-methods linear regression model was employed to predict bacterial counts using organism, time, and replicate culture values.

## Results

Time kill

**Figure 2:**
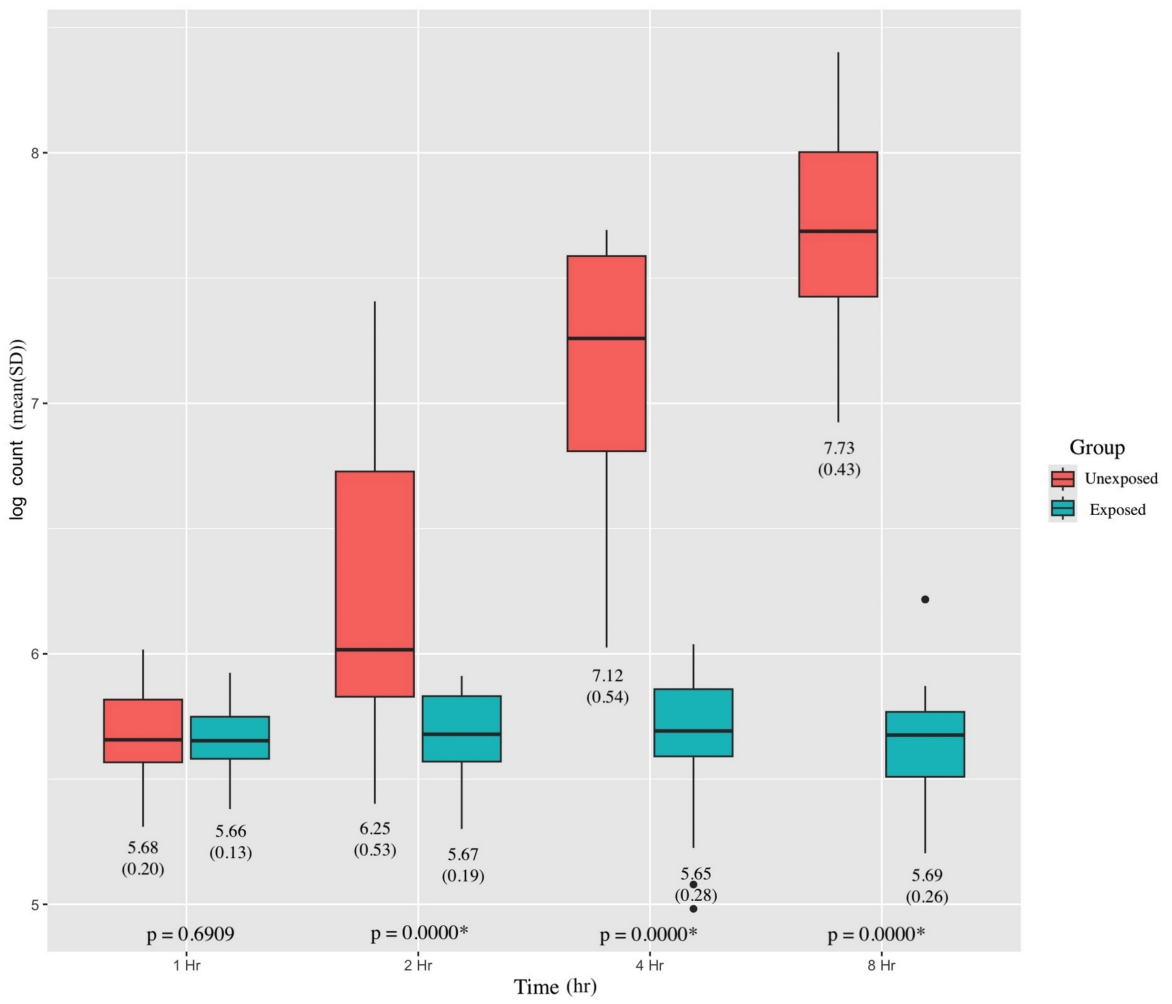
Time-kill log counts stratified by time and group Unpaired t-test, *p<0.05

We obtained 192 unique measurements from 48 colistin-sensitive replicates, representing eight organisms in the time-kill format. Figure 2 presents the mean (SD) log counts for the test and control groups at measured time points. The log count values were statistically lower in the colistin-exposed group at 2, 4, and 8 hours. Figure 2 also illustrates box plots of the log counts from the time-kill format at various time intervals, categorized by test or control groups.

Minimum inhibitory concentration

**Figure 3:**
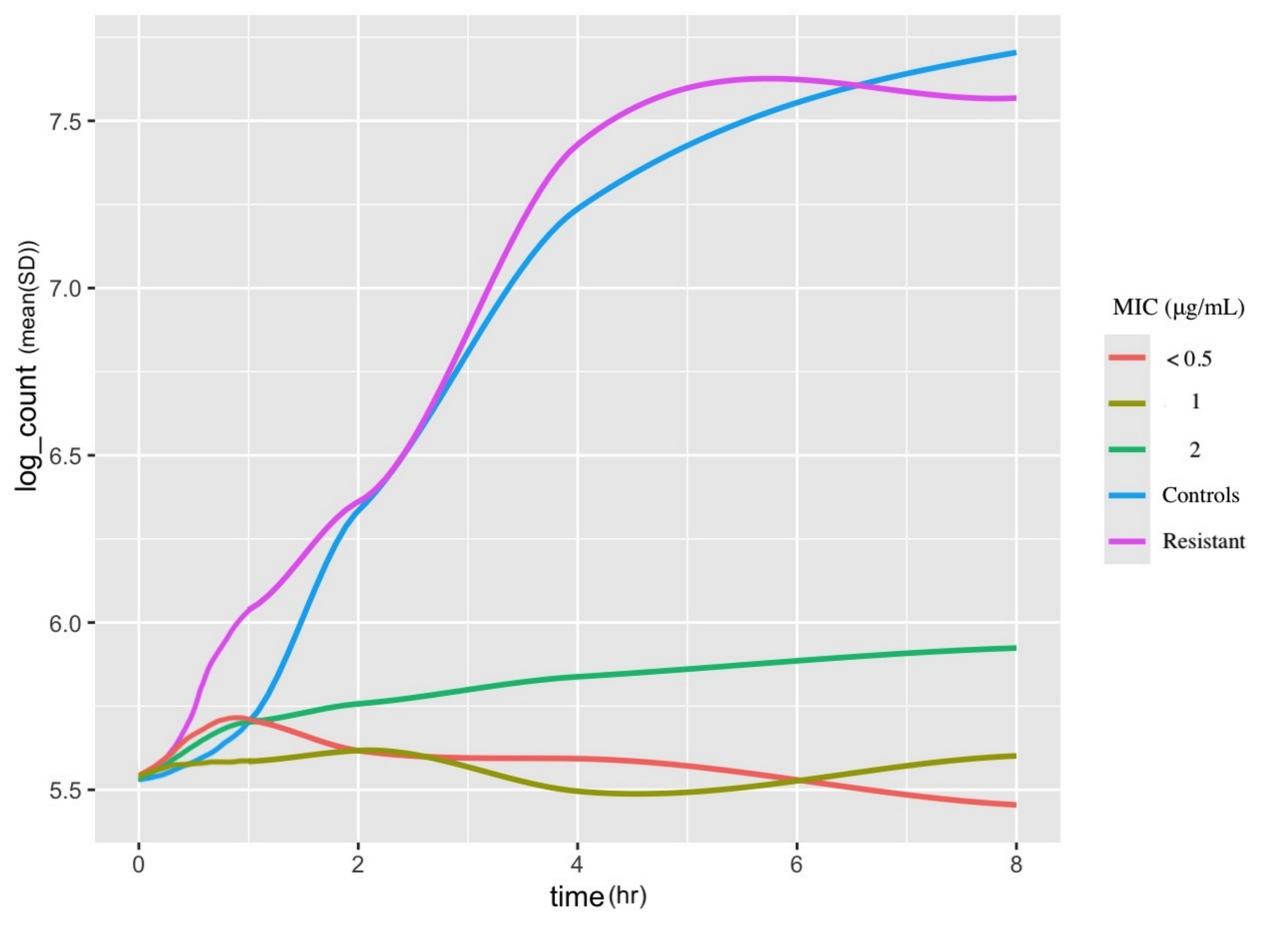
Time-kill log counts stratified by time and MIC MIC ≤ 0.5 µg/mL: *E. coli *ATCC 25922, *P. aeruginosa* (clinical) MIC =1 µg/mL: *P. aeruginosa* (clinical), *K. pneumoniae* (clinical), *A. baumannii* (clinical) MIC = 2 µg/mL: *E. coli* mcr-1, E. coli (clinical), *P. aeruginosa* (clinical) MIC ≥ 16 µg/mL: *K. pneumoniae* (clinical), *P. mirabilis *ATCC 12453 MIC, minimum inhibitory concentration

**Figure 4:**
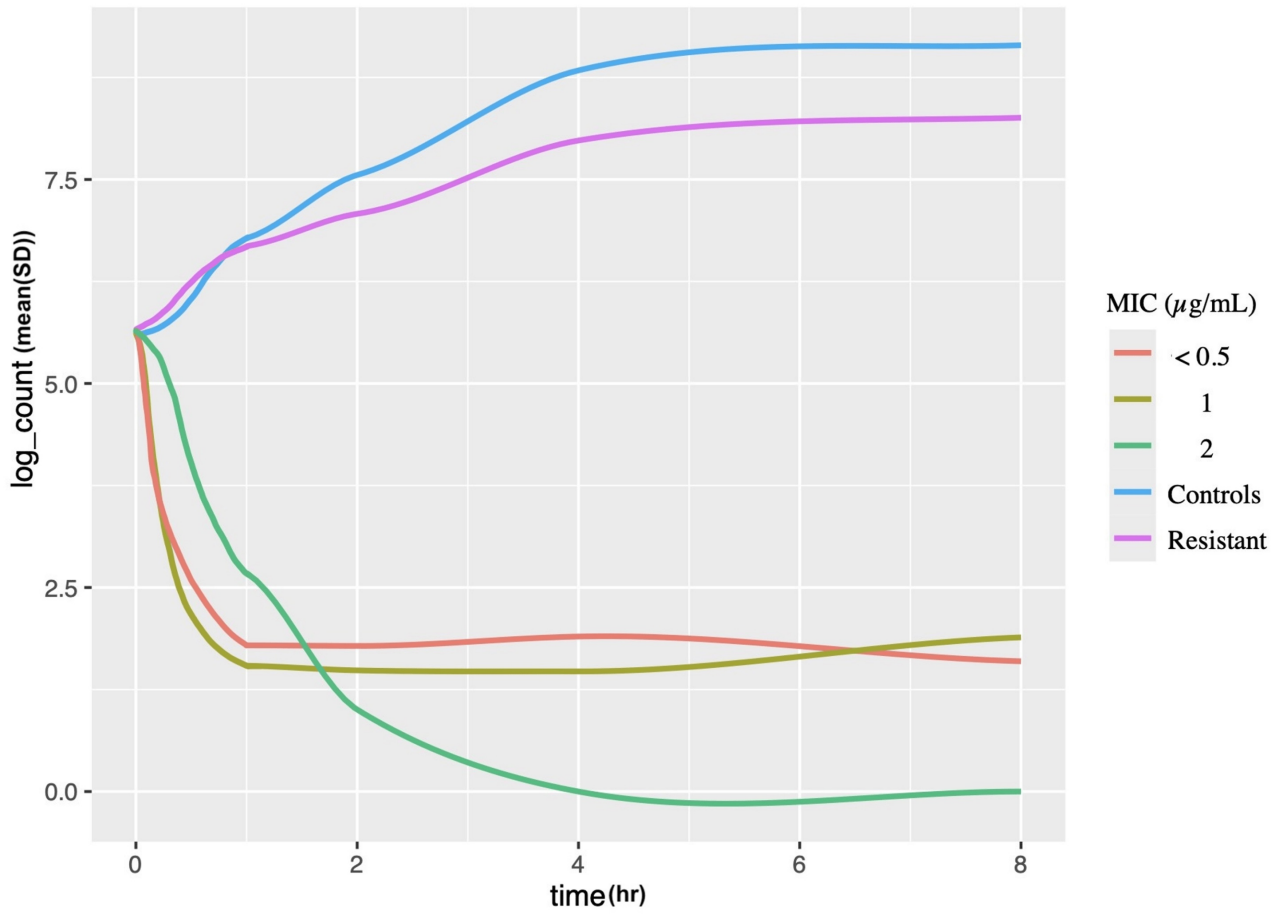
Cell-viability log counts stratified by time and MIC MIC ≤ 0.5 µg/mL: *E. coli* ATCC 25922, *P. aeruginosa* (clinical) MIC =1 µg/mL: *K. pneumoniae* (clinical), *A. baumannii *(clinical) MIC = 2 µg/mL: *E. coli* mcr-1, *P. aeruginosa* (clinical) MIC ≥ 16 µg/mL: *K. pneumoniae* (clinical), *P. mirabilis* ATCC 12453 MIC, minimum inhibitory concentration

Line plots in Figure 3 and Figure 4 depict log counts from the time-kill and cell-viability formats, respectively, across various time intervals, stratified by their MIC values. Significant overlap was observed in the confidence intervals of different MIC values. Consequently, bacterial cell counts could not be accurately predicted using MIC values in either the time-kill or cell-viability format.

Mixed linear regression

We fitted a linear mixed model (estimated using REML and Claptrap Optimiser) to predict the log count of cell viability with group and time, and 95% confidence intervals (CIs) and p-values were computed using a Wald t-distribution approximation. The model included groups (grouped by MIC) as random effects nested within the organism (and replicates) and time. Table 2 shows the results of the mixed effects linear regression analysis.

$$\log(\text{Count}) \sim \text{Group} \times \text{Time} + (\text{Group} \mid \text{Organism}/\text{R0}) + (\text{Group} \mid \text{Time})$$

Explanation:

$\log(\text{Count})$ represents the logarithm of the count variable.

$\sim$ indicates the model formula.

$\text{Group} \times \text{Time}$ includes both main effects and their interaction.

$(\text{Group} \mid \text{Organism}/\text{R0})$ implies random slopes for group nested within R0 within organism.

$(\text{Group} \mid \text{Time})$ represents random slopes for group across time.

**Table 2:** Adjusted mixed effects linear regression analysis for predicting log count *p<0.05

Predictors	Beta	Std. Beta	CI	p-value
(Intercept)	5.64	5.64	5.05–6.24	<0.001*
Group	0.01	0.01	-0.63 to 0.65	0.975
Time	0.28	0.17	0.16–0.40	<0.001*
Group x time	-0.28	-0.17	-0.40 to -0.15	<0.001*

The model explained a statistically significant and large proportion of variance (R^2^ = 0.889, p < 0.001). Group and time had statistically significant impacts on log counts in this model. The diagnostics indicate that the model accurately predicts log counts and has low levels of uncertainty (low collinearity). The linearity, homogeneity of variance, and normality of residuals were all found to lie within the reference ranges.

## Discussion

Colistin is a critical last-resort antibiotic for life-threatening multidrug-resistant bacterial infections due to the unavailability of a newer class of beta-lactam-beta-lactamase inhibitors, such as ceftazidime-avibactam and imipenem-relebactam. This is true particularly in low- and middle-income countries where either they are not marketed or the cost of treatment is very high. However, conventional colistin AST methods, such as BMD, are slow and labor-intensive. Newly recommended CBDE and CAD for colistin susceptibility testing are easy to perform but take 16-24 hours to yield susceptibility results and are not approved for all gram-negative strains such as *A. baumannii *complex [7,11]. Consequently, physicians empirically administer colistin at suboptimal doses while awaiting susceptibility results. This misuse of the drug creates selection pressure, leading to the emergence of colistin-resistant strains. Therefore, there is an urgent need to develop accelerated and automated assays to determine colistin susceptibility.

We conducted a time-kill and spread-plate assay to propose a time frame within which a microfluidics-based approach could provide results. We found a statistically significant difference between the bacterial counts of the exposed and unexposed groups of colistin-sensitive strains at 2, 4, and 8 hours in both the time-kill and spread-plate assays. While colistin-sensitive strains showed a decreased viable count, non-exposed and resistant strains (whether colistin-exposed or not) showed increased bacterial counts. Our study's mixed effects linear regression model explained 88.9% of the variability in bacterial count within our time-kill assay.

This model identified time and exposure to colistin as statistically significant predictors of colistin-sensitive bacterial cell counts, further reinforcing the strength of our findings. It explains a high proportion of variability, and “exposure to colistin” emerges as a statistically significant predictor for bacterial cell counts. This provides robust evidence in favor of using microfluidics to obtain colistin susceptibility results within 2 hours.

In this context, designing microfluidics-based indigenous devices holds promise for rapid AST assay. Microfluidics can monitor antibiotic resistance in real-time at a single-cell level by confining bacteria in gas-permeable microchannels and measuring their growth [12,14]. Droplet-based microfluidic methods can reliably predict antimicrobial susceptibility within 1 hour, and recent advances in genotypic-based microfluidic chips have lowered AST time to only 30 minutes [15-17].

These microfluidic techniques have eliminated the need for traditional organism culturing time. They can be integrated with methods, such as electrochemical, paper-based, and BMD-based tests, to create testing chips even for multiplex testing [18-21]. This can transform the current national AST landscape by automating the process, making it more affordable and faster while reducing sample and reagent waste. However, microfluidics-based AST for colistin is currently being attempted only sparingly. This study aimed to establish a foundation of evidence for developing microfluidic-based devices for colistin susceptibility testing. Our study drew a parallel between microfluidics and time-kill assays, commonly used to study the activity of a single/combination of antimicrobial agents against a bacterial strain over time [22-24].

Our study employed BMD, the gold standard for colistin susceptibility testing, ensuring accurate MIC determination validated against control strains. We used stringent growth and media controls and applied acridine orange staining in a Neubauer chamber, which enhanced clarity in time-kill assays. We included isolates with a wide MIC range for the time-kill assay and used linear mixed-effects modeling, which allowed for dynamic assessment of bactericidal activity. Comparative evaluation with Vitek® 2 highlighted important discrepancies, reinforcing the need for broader BMD implementation.

Our study has several limitations. The small sample size and limited number of isolates per species reduced generalizability and prevented a detailed assessment of heteroresistance. Time-kill assays were performed at a single concentration (4 μg/mL), which may not fully reflect pharmacodynamic variability across MIC ranges. The value of 4 μg/mL was chosen as it represents the breakpoint value for distinguishing resistance from the intermediate interpretive category [13,25]. The study was conducted using conventional formats, and aspects such as molecular characterization, and its implications, cross-validation, and correlation with clinical outcomes (i.e., in vivo relevance), were beyond its intended scope. While our findings support broader BMD adoption, their extrapolation across strains and dosing contexts should be cautiously approached. Importantly, this study offers a proof-of-concept for microfluidics-based AST, with clinical validation reserved for future work.

## Conclusions

In conclusion, our study provides evidence for developing rapid colistin susceptibility testing, which can detect susceptibility as early as 2 hours. By demonstrating significant differences in bacterial counts within 2 to 8 hours through time-kill and spread-plate assays, we establish a clear biological basis for accelerated testing methods. Future work should focus on using a wider range of strains, varying antibiotic concentrations, and validating findings on microfluidic prototypes to drive the development of efficient, real-time diagnostic tools.
